# A longitudinal investigation of caregiving and adolescent post-traumatic stress symptoms during COVID-19: evidence for high resting RSA as a susceptibility factor

**DOI:** 10.1017/S003329172400059X

**Published:** 2024-03-14

**Authors:** Linnea B. Linde-Krieger, Kristen L. Rudd, Alexandra S. Aringer, Tuppett M. Yates

**Affiliations:** 1 Department of Family and Community Medicine, College of Medicine, University of Arizona, Tucson, AZ, USA; 2 Department of Psychology, University of California, Riverside, CA, USA; 3 Department of Psychology, University of Colorado, Colorado Springs, CO, USA

**Keywords:** adolescence, caregiving, COVID-19, diathesis stress/dual risk, differential susceptibility/biological sensitivity to context, post-traumatic stress, psychophysiology

## Abstract

**Background.:**

Post-traumatic stress symptoms (PTSS) were the most frequently reported mental health concern for youth during COVID-19, yet variations in youth’s PTSS responses warrant empirical consideration. Features of the caregiving environment influence youth’s responses to environmental stressors, and youth’s parasympathetic nervous system regulation may qualify the magnitude and/or direction of these effects. This prospective investigation evaluated diathesis stress and differential susceptibility models of caregiving and parasympathetic influences on youth’s PTSS responses to COVID-19.

**Method.:**

Participants were 225 caregiver-youth dyads (youth 49.8% female at birth; 88.4% non-white) followed from childhood through adolescence and COVID-19. Youth’s resting respiratory sinus arrhythmia (RSA; *M*_*age*_ = 6.11, S.D. = 0.21), caregiving features (i.e. attachment security [youth *M*_*age*_ = 12.24, S.D. = 0.35] and caregiver internalizing psychopathology [caregiver *M*_*age*_ = 39.29, S.D. = 6.78]) were assessed pre-pandemic. Youth’s PTSS was assessed one year prior to the US COVID-19 pandemic (*M*_*age*_ = 14.24, S.D. = 0.50) and during the spring of 2020 at the height of the pandemic (*M*_*age*_ = 15.23, S.D. = 0.57).

**Results.:**

Youth’s PTSS increased during COVID-19. Youth with relatively high resting RSA evidenced the lowest PTSS when their caregiving environment featured high attachment security or low caregiver internalizing problems, but the highest PTSS when their caregiving environment featured low attachment security or high caregiver internalizing problems. In contrast, PTSS levels of youth with relatively low or average resting RSA did not differ significantly depending on attachment security or caregiver internalizing.

**Conclusions.:**

Results are consistent with a differential susceptibility hypothesis, wherein relatively high resting RSA conferred heightened sensitivity to caregiving environments in a for-better-and-for-worse manner during COVID-19.

The COVID-19 pandemic introduced a period of unprecedented stress and adversity for youth and families (Giannopoulou et al., 2021). The pandemic and attendant restrictions on daily life negatively impacted youth’s well-being, prompting acute and chronic stress reactions, heightened worry for the health and safety of self and others, and increased family stress (Guessoum et al., 2020; Raymond, Provencher, Bilodeau-Houle, Leclerc, & Marin, 2022). Post-traumatic stress symptoms (PTSS) were the most frequently reported mental health concern for children and adolescents during COVID-19 (Ma et al., 2021), with cross-sectional surveys documenting a wide range of clinical PTSS prevalence in youth (i.e. 14 to 69% across studies; Bhushan, Basu, & Ganai, 2022; Cao *et al*. 2022; Selçuk *et al*. 2021). Although prospective examinations of PTSS in response to COVID-19 are scarce, initial longitudinal evidence suggests that adolescents were more vulnerable to the effects of COVID-19 stress than younger children (Ma et al., 2021). Indeed, over a one-year period during COVID-19, adolescents’ PTSS increased while children’s PTSS declined (Raymond et al., 2022). This pattern is consistent with neurobiological evidence that adolescents experience rapid changes in limbic circuitry amidst relatively slower development of the prefrontal cortex, as well as marked declines in self-regulation capacities, greater sensitivity to environmental stressors, and increased vulnerability for the emergence of stress-related disorders (Beesdo-Baum et al., 2015; Powers & Casey, 2015).

## Caregiving and youth adjustment during COVID-19

COVID-19 mitigation measures (e.g. quarantines, school closures) disrupted youth’s normal routines and limited their access to social support (Golberstein, Wen, & Miller, 2020). At the same time, the quality of youth’s caregiving environments took on added significance as a result of stay-at-home orders, which increased youth’s exposure to primary caregivers and their reliance on them for support (Guessoum et al., 2020). Thus, in the context of COVID-19, features of the caregiving environment may be particularly important for understanding variability in adolescent’s PTSS responses.

Attachment theory suggests that youth seek contact and comfort from their caregivers in times of stress to varying degrees based on their representations of early caregiving relationships (Ainsworth, 1978; Schore & Schore, 2008). When early care is sensitive, attuned, and consistent, children are likely to develop a representation of the caregiver as a safe haven to which they can return for support and co-regulation when stressed (Bretherton & Munholland, 2008). Over time, children internalize repeated experiences of co-regulation with caregivers in ways that inform and guide their future navigation of stressful life events. For adolescents, secure attachment to a primary caregiver engenders confidence in both the caregiver and their own self-regulation capacities and fosters well-being and resilience to adversity (Moreira, Pedras, Silva, Moreira, & Oliveira, 2021; Rasmussen et al., 2019), including better socioemotional adaptation during COVID-19 (Coulombe & Yates, 2022). In contrast, when early caregiving is misattuned or inconsistent, children may be less able and willing to rely on caregivers for support and regulation in times of stress. When repeated experiences of successful co-regulation are lacking, children may develop insecure attachment styles that place them at increased risk for future maladaptation (Schore & Schore, 2008). In a similar fashion, caregiver psychopathology may place children at increased risk for later difficulties through disruptions in their caregiving environment and exposure to maladaptive cognitions and behavior (Goodman & Gotlib, 1999). These types of disturbances may undermine youth’s capacities to navigate stressful situations and contexts, such as the COVID-19 pandemic. Indeed, exposure to caregiver psychopathology (e.g. depression, anxiety) is a robust predictor of youth’s adjustment problems, including PTSS (Lombardo & Motta, 2008; Morris, Gabert-Quillen, & Delahanty, 2012).

In addition to direct influences of caregiving on youth’s adaptation, research demonstrates marked individual differences in adolescents’ sensitivity to caregiving behaviors and environments (Belsky, Bakermans-Kranenburg, & van IJzendoorn, 2007), including during COVID-19 (Miller et al., 2022). These findings underscore the need for efforts to identify moderators of caregiving effects on adolescents’ adjustment. Several theoretical models seek to explain prior evidence that individuals vary in whether and how much they are affected by environmental stressors and supports (Bakermans-Kranenburg & van Ijzendoorn, 2011; Caspi et al., 2002). *Differential susceptibility* (DS) and *biological sensitivity to context* (BSC) models hold that individual characteristics (e.g. genotype, physiology) may enhance sensitivity to both positive *and* negative environmental influences in a for-better-and-for-worse fashion (Belsky et al., 2007; Boyce & Ellis, 2005). Studies supporting these models show that the same individuals who are disproportionately impacted by environmental stressors also reap the most benefit from supportive environments (Blandon, Calkins, Keane, & O’Brien, 2008; Miller et al., 2013; Obradović, Bush, Stamperdahl, Adler, & Boyce, 2010). In contrast, *diathesis stress* or *dual risk* (DR) models predict that, while some individuals are more vulnerable to the negative effects of adversity, they do not fare better than others in positive or supportive environments (Belsky & Pluess, 2009; Paris, 2000). Studies supporting these models show that specific risk factors (e.g. blunted cortisol, Eisenlohr-Moul et al., 2018; behavioral inhibition, Lahat, Benson, Pine, Fox, & Ernst, 2016) predispose individuals to poorer functioning in the context of environmental stressors. However, other studies find only partial support for DS/BSC models or mixed support for DS/BSC and Diathesis Stress/DR models within the same sample. For example, some evidence suggests that youth with relatively well-developed capacities for parasympathetic regulation display better adjustment in supportive family contexts, but not worse adjustment in unsupportive family contexts (Eisenberg et al., 2012; Van der Graaff et al., 2016). Consistent with a Diathesis Stress/DR framework, Loginova and Slobodskaya (2021) found that parenting style interacted with child temperament such that fearful temperament predicted greater adjustment problems in the context of punitive parenting but not better adjustment in the context of positive parenting. However, in the same sample, these researchers also found evidence of DS/BSC, wherein children who scored higher on a measure of personality flexibility/plasticity showed more adjustment problems in the context of punitive parenting, but fewer problems in the context of positive parenting. Given equivocal findings, the current investigation evaluated competing hypotheses informed by DS/BSC and Diathesis Stress/DR theories to evaluate prospective relations from both positive and negative features of the pre-pandemic caregiving environment, namely attachment security and caregiver psychopathology, to adolescents’ PTSS during COVID-19 as moderated by their parasympathetic self-regulation.

## Resting respiratory sinus arrhythmia and parasympathetic self-regulation

Autonomic self-regulation encompasses parasympathetic (i.e. rest and digest) and sympathetic (i.e. fight or flight) nervous system processes that support flexible modulation of arousal in response to everyday events and traumatic experiences. Respiratory sinus arrhythmia (RSA) is a measure of heart rate variability that reflects parasympathetic influences on cardiac function (Beauchaine, 2001). Relatively high resting RSA reflects parasympathetic nervous system inhibition of cardiovascular activity to support active engagement with the social environment and flexible activation of an adaptive response (i.e. parasympathetic withdrawal) in contexts of stress (Del Giudice, Ellis, & Shirtcliff, 2011; Thayer & Lane, 2000). Conversely, relatively low resting RSA reflects poor parasympathetic control that may confer a vulnerability to insufficient or inflexible physiological mobilization in the face of stress (Beauchaine & Thayer, 2015; Campbell, Wisco, Silvia, & Gay, 2019). Research on the development of autonomic self-regulation shows that various exposures during and after pregnancy can influence offspring resting RSA (Glover, O’Connor, & O’Donnell, 2010; Martinez-Torteya et al., 2014), with most evidence indicating that prenatal, rather than postnatal, exposures shape resting RSA (Bush et al., 2017; Tibu et al., 2014). In typical development, resting levels of RSA increase rapidly across the first year of life as infants develop progressively coordinated responses to social stimuli (Porges & Furman, 2011) and stabilize during early childhood (Alkon, Boyce, Davis, & Eskenazi, 2011; Dollar et al., 2020).

## Resting RSA and the caregiving environment

According to polyvagal theory (Porges, 2007), RSA is uniquely tied to social regulation and adaptation. Theory and research suggest youth’s resting RSA may modify caregiving effects on development, but it is not yet clear whether youth with relatively high or low resting RSA are more sensitive to caregiving effects (Eisenberg et al., 2012). Some studies suggest that relatively high resting RSA may buffer against negative outcomes in contexts of problematic caregiving (e.g. child maltreatment, Gordis, Feres, Olezeski, Rabkin, & Trickett, 2010; family conflict, El-Sheikh, Harger, & Whitson, 2001). Likewise, other studies show that relatively low resting RSA constitutes a biological diathesis that exacerbates youth’s sensitivity to negative caregiving influences (e.g. parenting stress, Creavey, Gatzke-Kopp, & Fosco, 2018; family conflict, Katz & Gottman, 1997).

Consistent with DS/BSC models of development, other theorists posit that relatively high resting RSA may support youth’s active engagement with social environments in a way that sensitizes them to *both* positive and negative caregiving influences (Eisenberg et al., 2012; Ellis, Boyce, Belsky, Bakermans-Kranenburg, & van Ijzendoorn, 2011). When raised in a supportive environment, such as one that features secure caregiver-youth attachment, relatively high resting RSA promotes youth’s social engagement and attunement in ways that engender adaptive co-regulation with a sensitive and responsive caregiver (Conradt, Measelle, & Ablow, 2013). However, this same sensitivity to social influence may render youth with relatively high resting RSA disproportionately susceptible to the deleterious effects of negative caregiving environments, such as caregivers who endorse high levels of internalizing problems (Blandon et al., 2008). In this view, relatively high resting RSA promotes youth’s engagement with an anxious or depressed caregiver in ways that may undermine mental (McEwen, 2003) and physical (McEwen & Stellar, 1993) health.

## Study overview

This study leveraged psychophysiological and caregiving data obtained from a large and sociodemographically diverse sample of youth prior to the COVID-19 pandemic to examine whether and how resting RSA assessed at age 6 modified the effects of caregiving in early adolescence (i.e. age 12) on youth’s PTSS during the first phase of the COVID-19 pandemic in spring 2020 (i.e. age 15). Specifically, a priori-specified moderation models tested independent and interactive effects of positive (i.e. secure attachment) and negative (i.e. caregiver internalizing) caregiving features and childhood parasympathetic regulation (i.e. resting RSA) on adolescents’ PTSS during the COVID-19 pandemic above and beyond pre-pandemic PTSS (i.e. age 14) and sociodemographic covariates. We considered the effects of both adverse and supportive caregiving environments in line with recommendations to measure not only the presence and absence of adversity but also environmental support when evaluating DS/BSC theories (Ellis et al., 2011; Taylor, 2006).

Both Diathesis Stress/DR and DS/BSC models predict that relations between caregiving features and adolescents’ PTSS during COVID-19 would be moderated by youth’s resting RSA levels, albeit in different ways. The Diathesis Stress/DR model would expect ordinal interaction effects, in which youth with a putatively vulnerable pattern of parasympathetic nervous system functioning (e.g. relatively low resting RSA) in a negative caregiving context, such as a caregiver who endorses internalizing symptoms, would exhibit the highest levels of PTSS during COVID-19, whereas those with a more protective pattern of parasympathetic regulation (e.g. relatively high resting RSA) would be buffered against PTSS during COVID-19. However, DS/BSC models further predict that youth’s resting RSA will influence relations between positive caregiving features (e.g. attachment security) and PTSS responses to COVID-19. The DS/BSC model predicts disordinal (i.e. crossover) interaction effects in which youth with a putatively *sensitive* pattern of parasympathetic regulation (e.g. relatively high resting RSA) would differ from those with a less *sensitive* pattern of parasympathetic regulation (e.g. relatively low resting RSA) under both positive and negative caregiving conditions. In this view, youth with relatively high resting RSA and high attachment security would be expected to exhibit the lowest levels of PTSS during COVID-19, whereas youth with relatively high resting RSA and low attachment security would display the highest levels of PTSS during COVID-19. Similarly, youth with relatively high resting RSA in the context of elevated caregiver internalizing symptoms would be expected to evidence the highest levels of PTSS during COVID-19, whereas those with relatively high resting RSA but low caregiver internalizing psychopathology would evidence the lowest levels of PTSS during COVID-19.

## Method

### Participants

Participants were drawn from an ongoing longitudinal study of development among 250 caregiver-youth dyads. Youth participants (49.8% female at birth; 46.2% Latine, 17.8% Black, 11.6% white, 24.4% multiracial) were representative of the Southern California community from which they were recruited (U.S. Census Bureau, 2014). Primary caregivers were similarly diverse with respect to ethnicity and race (56% Latine, 19.1% Black, 19.1% white, 5.8% multiracial), and most were biological mothers (92.1%) who were married or in a committed relationship (80.4%). Nearly one-quarter (22.7%) of participating families resided below or near the federal poverty threshold (i.e. qualified for government aid, food stamps). Dyads (*N* = 225) who completed one or more study visits at ages 6 (*M*_*age*_ = 6.11, S.D. = 0.21), 12 (*M*_*age*_ = 12.24, S.D. = 0.35), 14 (*M*_*age*_ = 14.24, S.D. = 0.50), and/or 15 (*M*_*age*_ = 15.23, S.D. = 0.57) were included in these analyses. The vast majority of youth in the current sample remained with the same caregiver pre- and post-pandemic. Of participants who completed the age 15 assessment during COVID-19, only two experienced a change in caregiver from the time caregiving features (i.e. attachment security and caregiver internalizing) were measured at age 12.

### Procedure

Participants were recruited via flyers advertising a study on children’s learning and development. Caregivers completed a brief phone screening to ensure the child was (1) between 3.9 and 4.6 years of age at the time of recruitment, (2) proficient in English, and (3) not diagnosed with a developmental disability. At ages 6 and 12, dyads completed a three-hour laboratory assessment. At age 14, youth completed a telephone assessment of physical and mental health (including PTSS) approximately one year prior to the COVID-19 pandemic. At age 15, youth completed a similar online assessment at the start of the pandemic (i.e. spring 2020). Informed assent and consent were obtained from youth and their legal guardian, respectively. All procedures were approved by the human research review board of the participating university.

### Measures

#### Resting RSA

Mindware MW1000A ambulatory cardiography via Kendall Medi-Trace #133 spot electrodes collected measures of resting RSA at age 6. Spot electrodes were placed in a Lead II configuration on the child’s chest. Following a 5-min calibration period after initial electrode placement, resting RSA was measured during a non-challenging task (i.e. sorting pieces by shape and color). RSA data were filtered, extracted, and scored using Mindware’s HRV 3.0.10 analysis program (mindwaretech.com). Mindware’s algorithms calculated the variance in R-R wave intervals. RSA scores were calculated using the interbeat intervals on the ECG reading, respiratory rates derived from the impedance (i.e. dZ/dt) signal, and a specified RSA bandwidth range for 6-year-olds of 0.15 to 0.80 Hz (Bar-Haim, Marshall, & Fox, 2000). Further data cleaning included screening for outliers (i.e. >3S.D.) minute-by-minute in relation to each child’s data pattern and deleting data if more than 25% of the minutes were not scored. Consistent with prior studies (Alkon et al., 2011), RSA data were extracted in 30-s epochs across the 3-min baseline period.

#### Attachment security

The Behavioral Systems Questionnaire (BSQ; Furman & Wehner, 1999) measured youth’s perceptions of attachment security with their primary caregiver at age 12. The secure parent scale of the BSQ includes nine 5-point Likert items from 1 (*strongly agree*) to 5 (*strongly disagree*) capturing youth’s cognitions, affect, and behaviors related to attachment, caregiving, and affiliation with the primary caregiver (*α* = 0.84). The BSQ demonstrates high internal consistency and convergent validity with interview measures of attachment (Furman, Simon, Shaffer, & Bouchey, 2002).

#### Caregiver internalizing psychopathology

Caregivers reported internalizing psychopathology on the Brief Symptom Inventory (BSI; Derogatis & Fitzpatrick, 2004) when youth were 12 years old (caregiver *M*_*age*_ = 39.29, S.D. = 6.78). The BSI is an abbreviated form of the Symptom Checklist 90-Revised (Derogatis, 1990) with demonstrated reliability in clinical and community samples (Boulet & Boss, 1991; Derogatis & Melisaratos, 1983) and across diverse racial/ethnic groups (Hoe & Brekke, 2009). Items assessed how much symptoms of depression (6 items; *α* = 0.82), anxiety (6 items; *α* = 0.80), and somatization (6 items; *α* = 0.79) bothered caregivers in the past week on a 5-point scale from 0 (*not at all*) to 4 (*extremely*). Subscale scores were composited for use in these analyses (*rs* = 0.42 to 0.66).

#### Post-traumatic stress symptoms

We assessed PTSS on the widely used 118-item Achenbach Youth Self Report scale (YSR; Achenbach & Rescorla, 2001). The 14-item YSR post-traumatic stress problems subscale (PTSP; Achenbach & Rescorla, 2007; Winningham, Banks, Buetlich, Aalsma, & Zapolski, 2019) measured traumatic stress symptoms (e.g. ‘I can’t get my mind off certain thoughts,’ ‘I have nightmares’) one year prior to the COVID-19 pandemic when youth were 14 years old and again in spring 2020 when youth were 15 years old. Youth were instructed to rate the frequency of each symptom from 0 (*not true*) to 2 (*very or often true*) ‘during the past 6 months’ one year prior to pandemic, and ‘during the past 2 weeks’ at the start of the COVID-19 pandemic in spring 2020. We adjusted the reporting timeframe to the preceding two weeks during the age 15 assessment to ensure we captured symptomatology *during* the COVID-19 pandemic. Importantly, the YSR PTSP subscale conceptualizes these items as tapping post-traumatic stress symptoms but does not provide a diagnosis of post-traumatic stress disorder (PTSD), as diagnostic tools must measure both exposure to a specific traumatic event and symptomatology linked to the traumatic event. Therefore, to be consistent with prior studies and the intent of the PTSP subscale, we opted to conceptualize our outcome measure as PTSS severity rather than a diagnosis of PTSD. Prior research indicates that youth self-reports of PTSS provide increased accuracy over caregiver or teacher reports, and the PTSP subscale of the YSR has been used in diverse samples (Kirchner, Forns, Soler, & Planellas, 2014). Youth’s scaled *t*-scores on the PTSP subscale at ages 14 (*α* = 0.82) and 15 (*α* = 0.85) were used to indicate PTSS in these analyses.

#### Family income-to-needs

At age 12, caregivers reported all household income (e.g. salary, government assistance), and this was divided by the appropriate poverty threshold for household size and number of children under 18 in the home (U.S. Census Bureau, 2016).

#### Data preparation and analysis

Following preliminary descriptive and bivariate analyses, the R statistical program (R Core Team, 2022) evaluated regression models using the Full Information Maximum Likelihood (FIML) method of estimation to account for missing data, as supported by Little’s (1988) MCAR test, *χ*^2^(108) = 117.26, *p* = 0.26. FIML is among the most accurate and robust approaches for handling missing data and provides minimally biased parameter estimates and standard errors, as well as more accurate Type I error rates with greater statistical power (Enders & Bandalos, 2001; Myrtveit, Stensrud, & Olsson, 2001). Simulation studies show that FIML outperforms other methods of handling missing data under most conditions (Xiao & Bulut, 2020). In the current sample, all participants who completed any data wave were retained in the analyses as is required for the FIML method of estimation. Across time, 92% of dyads completed two or more assessments. *t* tests and *χ*^2^ analyses comparing dyads who completed one visit (*n* = 18) and those who returned for follow-up (*n* = 207) identified no statistically significant differences in age, sex, ethnicity-race, income, or any other study variables (all *ps* > 0.14). Data were missing due to attrition on resting RSA at age 6 in 12 cases (5.3%), on attachment security at age 12 in 32 cases (14.2%), on caregiver internalizing at age 12 in 30 cases (13.3%), on PTSS at age 14 (prior to COVID-19) in 65 cases (28.9%), and on PTSS at age 15 (during COVID-19) in 69 cases (30.7%). An additional 16 cases were missing resting RSA data due to computer malfunction (*n* = 11), electrode conduction problems (*n* = 2), outliers (*n* = 1), and task administration errors (*n* = 2). Youth reports of secure attachment were missing in 29 additional cases (12.89%) because the measure was not administered due to time constraints. *t* tests and *χ*^2^ analyses confirmed that participants who did not complete the BSQ measure due to time constraints were not statistically significantly different from those who did complete the BSQ measure on age, sex, ethnicity-race, income, or any other study variables (all *ps* > 0.33). An a priori power analysis (Faul, Erdfelder, Lang, & Buchner, 2007) found that power exceeded 0.80 to detect small to medium effects at a significance level of *a* = 0.05 in our regression model with two interaction terms and nine total predictor variables (i.e. interaction terms plus covariates). Additionally, in sensitivity analyses, all regression results described below replicated using raw data without FIML estimation, as well as in a sample omitting the two youth who experienced caregiver changes from age 12 to the age 15 COVID-19 follow-up.

Before conducting analyses, we developed an a priori analytic approach for assessing Diathesis Stress/DR and DS/BSC hypotheses using a multiple linear regression model with interaction terms. Predictor variables were mean centered to limit multicollinearity (Kraemer & Blasey, 2006). Significant interaction terms were probed by examining the effect of caregiving features on PTSS during COVID-19 at low (−1 S.D. below the mean), average, and high (+1 S.D. above the mean) levels of resting RSA. Regions of significance identified specific values of the predictors at which the slope between youth’s resting RSA and PTSS moved from significance to non-significance. As in previous work (Roisman et al., 2012), a proportion affected (PA) index quantified the degree to which interaction effects were more consistent with Diathesis Stress/DR (i.e. ordinal interaction) or DS/BSC (i.e. disordinal interaction) models. The PA index signifies the proportion of individuals who are differentially affected by the moderator. In other words, the PA index identifies the proportion of participants whose predictor scores fall below the crossover point of the interaction, which is the threshold at which the association between the moderator and the outcome changes meaning as a function of the predictor. The crossover point is calculated by dividing -*b*2 by *b*3, where -*b*2 is a negative transformation of the regression beta weight for the moderator (i.e. resting RSA) and *b*3 is the beta weight for the interaction term (i.e. resting RSA × secure attachment or resting RSA × caregiver internalizing). A PA index closer to 0 indicates most participants fall above the crossover point (i.e. ordinal interaction) and supports the Diathesis Stress/DR model because the effect is represented at high, but not low, levels of the predictor. A PA index closer to 0.50 indicates that roughly half the participants fall below and half fall above the crossover point (i.e. disordinal interaction) and supports the DS/BSC model because the effect is represented at both high and low levels of the predictor. Default conventions (Roisman et al., 2012) suggest that a PA index over 0.16, where >16% of cases (i.e. +1 S.D. above the mean) fall below the crossover point, may be interpreted as more consistent with DS/BSC than Diathesis Stress/DR hypotheses. The PA index assumes that predictor variables are normally distributed. In the current analyses, the distribution of caregiver internalizing psychopathology was non-normally distributed (skew = 2.74), as is often the case with symptom data. Thus, caregiver internalizing scores were log-transformed (post-transformation skew = 1.59) prior to regression analyses (Table 1).

## Results

### Descriptive and bivariate statistics

One year prior to the COVID-19 pandemic, 6.2% of adolescents in the current sample reported PTSS scores in the clinical range. In the weeks following the initial U.S. COVID-19 lockdown orders (i.e. spring 2020), youth’s PTSS scores increased significantly (*t*([134] = 2.66, *p* = 0.009) with reports in the clinical range more than doubling to 16.67%. PTSS scores did not differ significantly by adolescents’ sex assigned at birth or ethnicity-race ( *ps* > 0.09). Youth’s resting RSA was not significantly associated with PTSS scores prior to or during the pandemic, and PTSS scores at age 14 and during COVID-19 (i.e. age 15) were not significantly associated, indicating a change in rank order of adolescents’ PTSS scores during COVID-19. Youth’s resting RSA scores were positively and significantly associated with caregiver internalizing scores.

### Regression analyses

**Figure 1.**
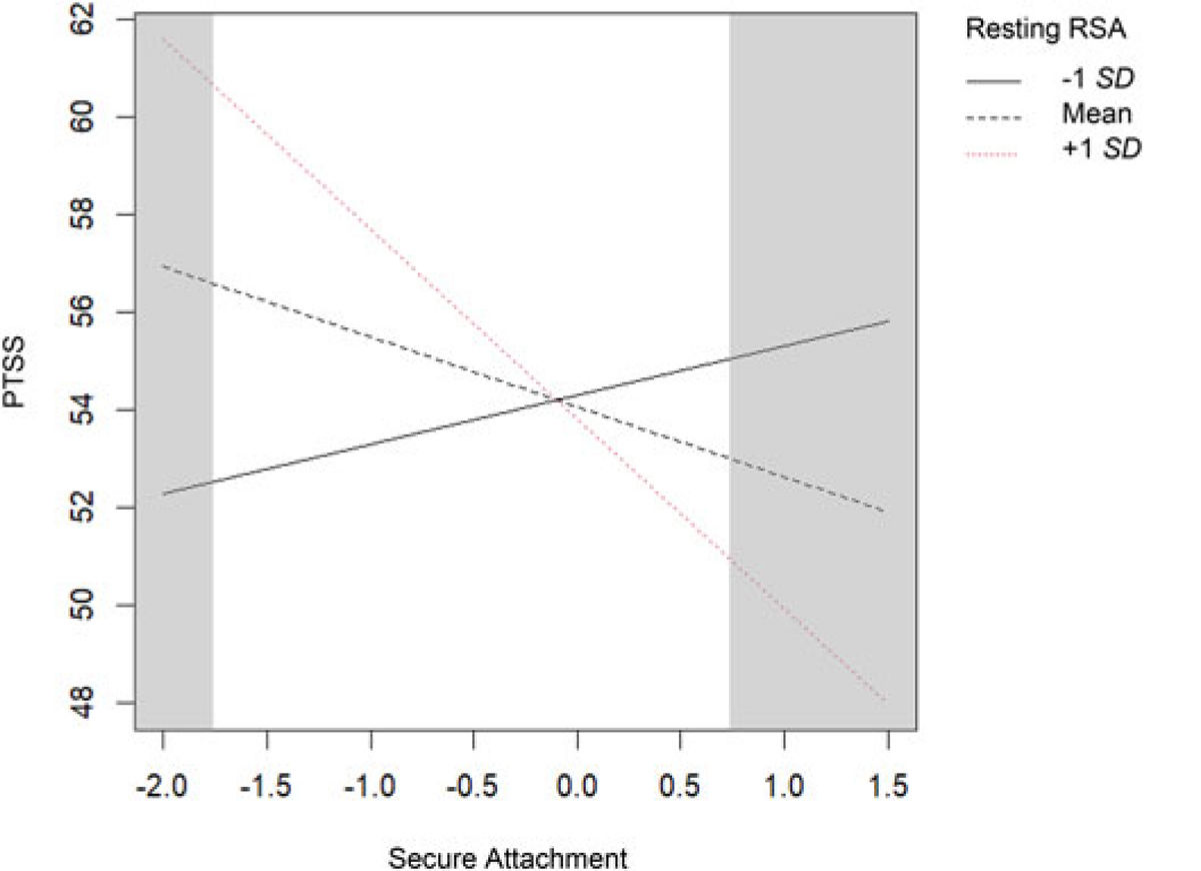
Youth resting RSA moderates the effect of secure attachment prior to COVID-19 on youth PTSS in response to COVID-19. *Note:* Predictor variables were mean centered. Effects are shown at mean and ±1 S.D. above and below the mean on resting RSA (mean centered range = −3.1 to 2.8). Simple slope analyses showed that secure attachment scores were negatively related to youth PTSS during COVID-19 when youth resting RSA was relatively high (*b* = −0.32, *p* = 0.01), but not when youth resting RSA was average (*b* = −0.11, *p* = 0.18) or relatively low (*b* = 0.09, *p* = 0.57). Shaded areas represent regions of significance where secure attachment scores (mean centered range = −1.66 to 0.89) outside the interval [−1.55 to 0.74] predicted PTSS during COVID-19 for youth with relatively high resting RSA.

A summary of the regression model is presented in Table 2. Main effects were not significant, indicating that neither secure attachment (*B* = −1.368 [−2.788 to 1.709], *p* = 0.191) nor caregiver internalizing (B = 2.917 [−2.127 to 7.960], *p* = 0.257) scores before the pandemic predicted adolescents’ PTSS during COVID-19 beyond pre-pandemic PTSS. The covariance of secure attachment and caregiver internalizing psychopathology was also nonsignificant (*σ* = −0.038 [−0.028 to 0.017], *p* = 0.630). However, there was a significant interaction between secure attachment and youth’s resting RSA on adolescents’ PTSS during COVID-19 after controlling for pre-pandemic PTSS and sociodemographic covariates (*B* = −2.449 [−4.448 to −0.449], *p* = 0.016). Probing conditional effects and regions of significance revealed that adolescents with relatively high resting RSA (+1 S.D. above the mean) evidenced the lowest PTSS scores when they reported higher secure attachment scores; however, adolescents with relatively high resting RSA who reported lower secure attachment scores evidenced the highest PTSS scores during COVID-19 (Fig. 1). Attachment security was not significantly related to adolescents’ PTSS during COVID-19 when youth exhibited average or relatively low resting RSA (−1 S.D. below the mean). The PA index for this interaction indicated that 38.4% of adolescents fell below the crossover point, which supports a DS/BSC hypothesis. A post-hoc power analysis with a significance level of *a* = 0.05 confirmed that power to detect the significant interaction of secure parenting and resting RSA exceeded 0.90 in this sample (Aberson, 2019; Baranger et al., 2022).

**Figure 2.**
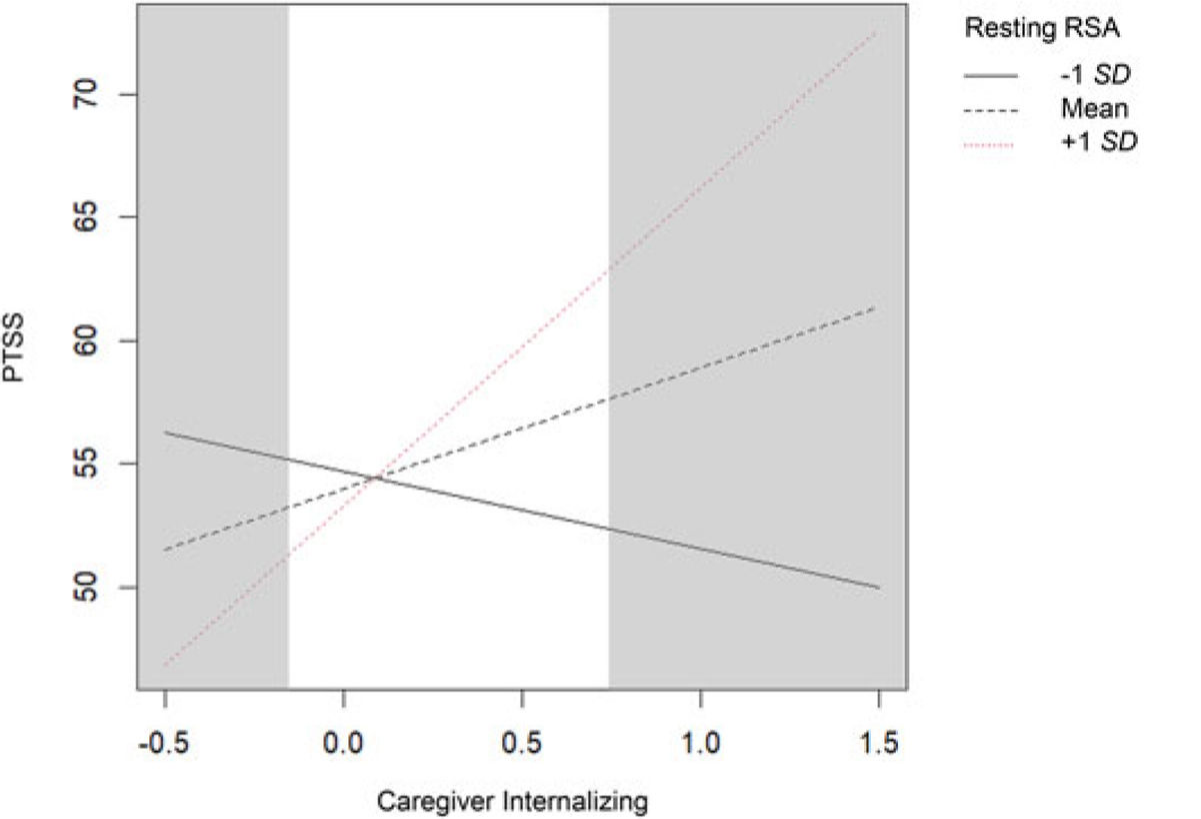
Youth resting RSA moderates the effect of caregiver internalizing symptoms prior to COVID-19 on youth PTSS in response to COVID-19. *Note:* Predictor variables were mean centered. Effects are shown at mean and ±1 S.D. above and below the mean on resting RSA. Simple slope analyses showed that caregiver internalizing symptoms were positively related to youth PTSS during COVID-19 when youth resting RSA was relatively high (*b* = 0.32, *p* = 0.002), but not when youth resting RSA was average (*b* = 0.08, *p* = 0.34) or relatively low (*b* = −0.15, *p* = 0.25). Shaded areas represent regions of significance where caregiver internalizing scores (mean centered range = −0.22 to 0.92) outside the interval [−0.13 to 0.72] predicted PTSS during COVID-19 for youth with relatively high resting RSA.

Youth’s resting RSA also moderated the association between caregiver internalizing psychopathology and adolescents’ PTSS during COVID-19 (*B* = 7.990 [2.002–13.978], *p* = 0.009). Probing conditional effects and regions of significance revealed that adolescents with relatively high resting RSA evidenced the highest PTSS scores when their caregivers reported greater internalizing psychopathology, but the lowest PTSS scores when their caregivers reported low or no internalizing psychopathology (Fig. 2). Caregiver internalizing was not significantly related to adolescents’ PTSS during COVID-19 for youth with average or relatively low resting RSA. The PA index for this interaction indicated that 65.6% of adolescents fell below the crossover point, which supports a DS/BSC hypothesis. A post-hoc power analysis confirmed that power to detect the significant interaction of caregiver internalizing and resting RSA exceeded 0.93 in this sample. Additionally, in a post-hoc evaluation, all regression results replicated without covariates included in the model.

## Discussion

This longitudinal study offers a novel evaluation of adolescents’ PTSS responses to COVID-19 as influenced by caregiving features (i.e. secure attachment, caregiver internalizing psychopathology) in early adolescence and youth’s parasympathetic self-regulation (i.e. resting RSA). Results demonstrated significant interaction effects of both positive and negative caregiving features with youth’s resting RSA in predicting adolescents’ PTSS responses to COVID-19. Evaluating interaction effects via simple slopes, regions of significance, and PA indices revealed a consistent pattern of results, such that youth with higher resting RSA appeared more susceptible to caregiving effects in a for-better-and-for-worse manner. Notably, these effects held for both youth-reported (i.e. secure attachment) and caregiver-reported (i.e. caregiver internalizing) measures of caregiving environments, and after controlling for prior levels of youth’s PTSS and sociodemographic covariates. Results are consistent with DS/BSC models of sensitivity to environmental influence and point to promising targets for prevention and intervention.

Adolescence is a sensitive developmental period for the emergence of psychological disorders (Powers & Casey, 2015), perhaps especially in stressful contexts (Larson, Moneta, Richards, & Wilson, 2002) such as the COVID-19 pandemic (Park et al., 2020). In the current sample, adolescents reported higher mean levels of PTSS during COVID-19 as compared to one year before the pandemic, and PTSS scores in the clinical range more than doubled during COVID-19. These findings are consistent with prior research showing increases in stress-related psychopathology among adolescents during COVID-19 (Golberstein et al., 2020; Ma et al., 2021; Raymond et al., 2022). Pandemic- and quarantine-related stress may have initiated or exacerbated youth’s PTSS during a time when adolescents experienced reduced access to mental health services and fewer contacts with extrafamilial adults who could recognize and respond to their distress.

Theory suggests that caregiving features are important influences on youth’s adjustment during times of stress (Lombardo & Motta, 2008; Morris et al., 2012), and that youth who can rely on caregivers for successful co-regulation may be protected against stress-related psychopathology (Moreira et al., 2021; Rasmussen et al., 2019; Schore & Schore, 2008). Interestingly, in the current sample, neither secure attachment nor caregiver internalizing problems prior to the pandemic exerted significant direct effects on youth’s PTSS in response to COVID-19. Non-significant direct effects of caregiving features on youth’s PTSS underscore the probabilistic nature of development wherein compounding factors (e.g. caregiving and physiology) may be related to increased probabilities of certain outcomes. The current findings speak to those probabilities while illuminating factors, such as parasympathetic regulation, that may alter them. Indeed, this study identified youth’s resting RSA as a modifier of the impact of caregiving features on youth’s PTSS during COVID-19. Following a DS/BSC hypothesis, caregiving effects varied across youth, with the most positive *and* most deleterious effects experienced by youth with relatively high resting RSA, which may confer heightened physiological attunement to both positive and negative social environments (Ellis et al., 2011).

During periods of heightened stress, such as COVID-19, physiological regulation may take on increased importance as youth attempt to navigate prolonged uncertainty and significant disruptions to daily life (Porges, 2020). In the context of stable and supportive caregiving relationships, youth with relatively well-developed capacities to mount an adaptive physiological response to stress may be especially well-equipped to harness the protective power of a secure parent-child relationship in the face of challenge (Obradović et al., 2010). Indeed, adolescents with relatively high resting RSA and more favorable caregiving environments (i.e. high attachment security or low caregiver internalizing) appeared better able to navigate COVID-19 stressors with less PTSS. At the same time, however, these physiologically sensitive youth with relatively high resting RSA reported the highest levels of PTSS when their caregiving environment was characterized by low attachment security or high caregiver internalizing psychopathology. Youth who felt less securely attached may have felt less able to turn to caregivers for support amidst the stress and threats of COVID-19. At the same time, caregivers with relatively elevated internalizing symptoms may have experienced greater difficulty regulating their own emotions and behaviors during COVID-19 in ways that undermined their support for their adolescent.

The current findings are consistent with previous studies showing that youth with relatively high resting RSA evidence heightened sensitivity to features of the caregiving environment including caregiver-child attachment security (Conradt et al., 2013) and caregiver psychopathology (Blandon et al., 2008). However, they contrast with other evidence suggesting that low resting RSA reflects a biological diathesis to adversity (El-Sheikh, 2005; McLaughlin, Rith-Najarian, Dirks, & Sheridan, 2015). These divergent findings may reflect differences in the features examined across studies (e.g. attachment security *versus* community violence) and/or the severity of adversity (e.g. insensitive care *versus* maltreatment). Under more severe conditions (e.g. child maltreatment, high community violence; McLaughlin et al., 2015), less responsive parasympathetic regulation (i.e. relatively low resting RSA) may represent a diathesis that magnifies negative caregiving effects. Importantly, the current study addressed a major limitation of prior research on parasympathetic regulation and youth’s adjustment, namely the overwhelming emphasis on negative environmental influences, by responding to calls for studies that examine the full continuum of environmental influences (Ellis et al., 2011; Taylor, 2006).

### Limitations

This study featured important strengths in its evaluation of both positive and negative caregiving features using multiple informants in a demographically and socioeconomically diverse sample of youth who were followed from childhood through adolescence and the COVID-19 pandemic. However, several limitations qualify these findings while pointing to promising directions for future research. First, participants in the current sample were drawn from Southern California, which may introduce selection bias and limit the generalizability of study findings. Second, as in all longitudinal studies, data were missing due to attrition such that between 5% and 31% of cases were missing data on any given variable across study waves. Importantly, regression results using the FIML method of estimation replicated in sensitivity analyses using the raw data.

Third, resting RSA was assessed at age 6, which was several years prior to the onset of the COVID-19 pandemic. Although prior work shows that childhood resting RSA is a relatively stable indicator of later physiological regulation (Alkon et al., 2011; Dollar et al., 2020), the remote nature of COVID-19 data collection precluded follow-up RSA measures to confirm the stability of resting RSA at age 15. Fourth, burgeoning work suggests that exposure to various life events during pregnancy and infancy can influence offspring physiology (Bush et al., 2017; Tibu et al., 2014). Although these early exposures may provide insight into how some youth develop certain regulatory patterns (e.g. high resting RSA *versus* low resting RSA), the current study did not evaluate prenatal or early childhood exposures, and, therefore, we were unable to control for this in the current analyses. Further, we focused on resting RSA as an indicator of parasympathetic regulation, but future research should examine both branches of the autonomic nervous system (i.e. incorporating pre-ejection period measures of sympathetic regulation; Marshall, 2013; Rudd & Yates, 2018), multiple phases of the stress response (i.e. reactivity and recovery; Rudd, Alkon, & Yates, 2017), and dynamic interrelations of physiological processes across time (Gatzke-Kopp, Benson, Ryan, & Ram, 2020; Rudd & Yates, 2020).

Fifth, several measurement issues should be considered when evaluating these findings. For example, this study employed a validated youth self-report measure of attachment security that demonstrates convergent validity with interview measures of attachment (Furman et al., 2002), but interviews are considered the gold standard for assessing attachment representations beyond childhood (Target, Fonagy, & Shmueli-Goetz, 2003). Our measure of caregiver internalizing was limited to symptoms during the past week, which may not have captured caregivers’ broader or trait-like internalizing problems. Similarly, our measure of youth’s PTSS captured traumatic stress symptoms but did not assess exposure to specific traumatic events and was not a diagnostic tool. Youth were asked to consider their symptoms *during the COVID-19 pandemic* while responding to items capturing traumatic stress reactions. We framed the instructions this way with the intent to measure PTSS in response to COVID-19; however, the lack of an index event beyond the COVID-19 pandemic is a limitation that precluded classification of stress-related disorders, such as PTSD. Future research should add to our understanding of the complex and multilevel influences on youth’s stress reactions, which may or may not eventuate in psychopathology, in highly stressful contexts such as the COVID-19 pandemic. For example, features of the caregiving environment, such as attachment security and caregiver psychopathology, may not be fully independent. Future research should consider higher-order interactions of multiple caregiving features and youth’s physiological regulation on youth’s stress reactions.

Finally, future research will benefit from similar tests of moderation across varying degrees of contextual stress. Interestingly, in the current sample, the strength of the bivariate association between resting RSA and youth’s PTSS decreased from pre- to post-COVID-19. This may reflect the heightened salience of RSA as a moderator of caregiving influences during the COVID-19 stressor. Indeed, a post-hoc test of the obtained regression model using pre-pandemic PTSS as the outcome revealed no significant interaction effects between caregiving features and resting RSA. Together, these differential patterns across contextual strain suggest that both physiological regulation and caregiving influences were heightened for youth during the COVID-19 pandemic, such that the moderating influence of RSA on caregiving effects strengthened as broader contextual risk increased. During the initial weeks of the COVID-19 pandemic, youth experienced unprecedented life disruptions, including reduced social interaction and access to peers and teachers, increased time spent in the home environment with caregivers, heightened health and safety concerns, and potentially increased exposure to caregiver stress. For many youth, COVID-19 represented a unique period of stress that may have magnified the salience of both youth’s self-regulation capacities and environmental effects on youth’s PTSS responses.

### Clinical implications

Notwithstanding the need for studies to replicate and expand the obtained findings while considering additional covariates (e.g. financial stress, relationships beyond the primary caregiver), this study has important implications for assessment and intervention in contexts of stress. Stressors, such as the COVID-19 pandemic, can instantiate psychopathological pathways that place adolescents at increased risk for a range of negative outcomes. However, this study demonstrates that efforts to promote supportive caregiving *and* positive physiological self-regulation in stressful environments may stem these pathways. Results highlight the value of multilevel interventions informed by biopsychosocial models of health (Bolton & Gillett, 2019). This multi-system framework emphasizes the interplay between biological, psychological, and environmental contributors to mental and physical health. For example, providers may draw on approaches from biomedical science, cognitive and behavioral neuroscience, behavioral skills training, and family systems theories when assessing and responding to adolescents’ distress.

In addition to caregiver and family-level interventions to strengthen secure attachment and support caregiver mental health, promoting adolescents’ self-regulation skills may help prevent or minimize traumatic stress reactions. Specifically, mounting evidence supports the effectiveness of mindfulness (Voss, Bogdanski, Langohr, Albrecht, & Sandbothe, 2020) and biofeedback (Goessl, Curtiss, & Hofmann, 2017) for promoting adaptive physiological regulation. Moreover, multi-level interventions, such as Attachment and Biobehavioral Catch-up (ABC; Dozier, Roben, Caron, Hoye, & Bernard, 2018), which is focused on supporting positive caregiving interactions to increase child regulation abilities (including parasympathetic regulation), have been shown to be effective (Grube & Liming, 2018). Future research is needed to determine if screening and assessment of resting RSA, as opposed to other measures of physiological or emotional regulation, will be beneficial for treatment planning and targeted intervention. Moreover, regulatory metrics may be fruitful avenues to identify youth – and their caregivers – who would benefit most from targeted interventions to increase environmental support. Importantly, ongoing research and policy efforts are needed to increase access to interventions while reducing burdens associated with such interventions for families with limited resources. Overall, the current findings enhance our understanding of both caregiving and physiological contributors to stress and resilience during the COVID-19 pandemic.

## Figures and Tables

**Table 1.** Descriptive and bivariate correlations for all study variables

Variable	*N*	*M* (S.D.)	1	2	3	4	5
1. Income-to-needs	198	2.37 (1.51)	–				
2. Pre-pandemic PTSS	160	53.21 (5.17)	−0.015	–			
3. Resting RSA	197	6.74 (0.99)	−0.033	0.143	–		
4. Secure attachment	164	4.11 (0.60)	0.014	−0.164	0.033	–	
5. Caregiver internalizing	195	0.29 (0.40)	−0.082	0.155	0.166*	−0.037	–
6. PTSS during COVID-19	156	55.58 (7.40)	−0.008	0.008	−0.043	−0.146	0.140

Note:

* *p* < 0.05. PTSS, post-traumatic stress symptoms; RSA, respiratory sinus arrhythmia.

**Table 2.** Regression analysis predicting youth post-traumatic stress symptoms during COVID-19

	*B*	S.E.	95% CI [LL, UL]	*p*
Ethnicity-race (Latine = 1)	−0.540	1.147	[−2.788 to 1.709]	0.638
Sex at birth (Female = 1)	1.247	1.135	[−0.977 to 3.471]	0.272
Income-to-needs	0.007	0.383	[−0.745 to 0.758]	0.986
Pre-pandemic PTSS	−0.006	0.123	[−0.247 to 0.236]	0.963
Resting RSA	−0.542	0.638	[−1.792 to 0.709]	0.396
Secure attachment	−1.368	1.047	[−3.420 to 0.684]	0.191
Caregiver internalizing	2.917	2.573	[−2.127 to 7.960]	0.257
Secure attachment × RSA	−2.449	1.020	[−4.448 to −0.449]	0.016*
Internalizing × RSA	7.990	3.055	[2.002–13.978]	0.009**

Note:

* *p* < 0.05;

** *p* < 0.01. PTSS, post-traumatic stress symptoms; RSA, respiratory sinus arrhythmia. Overall model fit was good (χ^2^(9) = 15.81, *p* = 0.07; CFI = 1.00; TLI = 1.00; RMSEA < 0.01, *R*^2^[PTSS during COVID-19] = 0.13). LL and UL indicate the lower and upper limits of the confidence interval.
